# Comparison of Clinical Features and Hemodynamics of Single Ventricle Patients With and Without Interventional Closure of Veno-Venous or Aortopulmonary Collaterals

**DOI:** 10.3390/jcdd12110444

**Published:** 2025-11-13

**Authors:** Helena Link, Alessia Callegari, Walter Knirsch, Oliver Kretschmar, Daniel Quandt

**Affiliations:** 1 Division of Pediatric Cardiology, Pediatric Heart Centre, University Children’s Hospital Zurich, 8008 Zurich, Switzerland; helena.link@kispi.uzh.ch (H.L.); walter.knirsch@kispi.uzh.ch (W.K.); oliver.kretschmar@kispi.uzh.ch (O.K.); daniel.quandt@kispi.uzh.ch (D.Q.); 2 Children’s Research Centre, University of Zurich, 8008 Zurich, Switzerland

**Keywords:** veno-venous collaterals, APCs, Fontan, single ventricle, embolization, closure

## Abstract

In single ventricle (SV) patients, veno-venous (VVCs) and major aortopulmonary collaterals (APCs) are common. We aim to determine the differences between patients with and without interventional closure of VVCs or APCs in this single-center, retrospective analysis of 135 SV patients (2006–2021). Anatomical, surgical and clinical features, hemodynamics and PA dimensions were compared. VVC closure was performed in 34 (25%) patients. VVC closure was associated with comprehensive stage I + II (*p* = 0.05), left PA patch procedure (*p* = 0.04), Fontan fenestration (*p* < 0.001), PA pressure >/= 16 mmHg pre-BCPC (*p* = 0.04) and >/= 15 mmHg pre-TCPC (*p* = 0.021), lower saturation pre-TCPC (*p* = 0.04) and smaller PAs (Nakata *p* = 0.03, total lower lobe index *p* = 0.001). Patients with VVC closure had a longer hospitalization post-BCPC (*p* = 0.008) and post-TCPC (*p* = 0.04) and longer ICU time post-BCPC (*p* = 0.04). APC closure was performed in 53 (39%) patients. Male sex (*p* = 0.001), Norwood I procedure (*p* = 0.04), younger age at BCPC (*p* = 0.02), higher end-diastolic pressure pre-TCPC (*p* = 0.04), lower oxygen levels pre-BCPC (*p* < 0.001) and smaller PAs (Nakata *p* = 0.08; McGoon *p* < 0.001) were related to APC closure. Patients with APC closure had a longer hospital (*p* < 0.001) and ICU (*p* = 0.005) stay post-TCPC. Significant VVC and APCs are linked to poorer PA growth, worse hemodynamics, lower sats and prolonged hospital/ICU stays, highlighting the need for targeted attention.

## 1. Introduction

Surgical palliation of SV patients is performed in up to three stages to separate the pulmonary and systemic blood flow. Stage I palliation is performed in the newborn period (if needed) to balance the pulmonary and systemic blood flow. After a few months, a bidirectional cavopulmonary connection (BCPC) is performed to partially separate the pulmonary circulation, while a total cavopulmonary connection (TCPC) then results in a complete oxygenation of the venous blood [1]. After TCPC, blood circulation relies entirely on a passive venous return to drive blood flow through the pulmonary arteries without the assistance of a sub-pulmonary ventricle. A common problem in single ventricle patients after BCPC and TCPC is the development of different types of collateral vessels, due to various reasons [2,3,4].

In the case of a single ventricle diastolic or systolic failure or obstruction in the BCPC or TCPC pathway, postcapillary end diastolic pressure (EDP) of the systemic ventricle or pulmonary artery pressure (PAP) raises disproportionally [5,6]. For pressure relief and a reduction of the transpulmonary gradient, veno-venous collaterals (VVCs) form by dilation of pre-existing embryologic venous channels. VVCs originate from the systemic veins and either drain into the pulmonary (PV) or the systemic venous (SV) system. The reported incidence of VVCs in single ventricle patients varies from 14 to 31% [6,7,8]. After BCPC, the systemic venous to systemic venous collaterals lead to a run-off of blood flow into the low-pressure circulation of the inferior vena cava (IVC) [2,9] and therefore to pressure reduction. Thus, the effective pulmonary blood flow is reduced and, together with the right-to-left shunt via the VVCs, this might lead to systemic arterial desaturation [10]. In patients with reduced oxygen saturation or exercise-related hypoxia, the treatment option is an interventional closure to increase systemic oxygen saturation, although the effect of embolization is controversially discussed, mainly because of the risk of recurrence. Additionally, VVCs increase the risk of paradoxical thromboembolism [10,11]. Small collateral vessels can be easily closed using metal spirals (coils), whereas large vessels may require placement of intravascular devices [12].

Aortopulmonary collaterals (APCs) are persistent/reopened segmental arteries or newly formed vessels which connect the aorta or aortic branches with the pulmonary artery vessel bed. They form in conditions with compromised pulmonary blood flow in order to increase the pulmonary circulation and oxygen saturation [13,14]. Hypoxia is known to stimulate the release of angiogenic factors, promoting the growth of new vessels, including APCs, to enhance oxygen delivery to the lungs [15]. A high pulmonary vascular resistance also encourages the formation of APCs, to ensure some degree of pulmonary perfusion.

The reported prevalence of these collaterals in single ventricle patients varies between 18 to 85% [16,17]. APCs contribute to the development of high pulmonary pressures, which in turn imposes an additional workload and hemodynamic burden on the SV [2,14]. Transcatheter closure is mostly performed (using coils or occlusion devices) in patients with elevated SV end diastolic pressure or pulmonary pressure, but also prior to surgical procedures, in order to avoid massive intraoperative backflow to the pulmonary arteries [6,17,18]. The effectiveness of APC closure is controversially discussed, as new collaterals are reported to be very likely to develop [17].

In this study, we describe a cohort of SV patients that underwent the interventional closure of VVC and/or APCs. We sought to determine the differences in patients with and without VVCs or APCs closure in relation to patient characteristics, clinical course, such as length of intensive care unit (ICU) or hospital stay, hemodynamics, oxygen levels and pulmonary artery dimensions.

## 2. Materials and Methods

### 2.1. Study Design and Patients’ Selection

This was a single-center, retrospective, longitudinal analysis of 135 single ventricle patients (2006–2021) who underwent at least two of the three steps of Fontan palliation. All SV patients born in our center between March 2006 and January 2021 who were initially directed toward SV palliation were included. All patients underwent BCPC and 131 of the patients underwent TCPC. Four (4/135) patients have not received TCPC yet and in two cases (2/135) we cannot provide surgery details on TCPC, as it was performed in a different center. At least two cardiac catheterizations were performed in each patient (one before BCPC and one before TCPC), in accordance with our institutional standard. The cardiac catheter team and incidence of closure have not changed in the last 15 years, which allows the comparison within this cohort. Demographic data, underlying congenital heart disease, preceding congenital cardiac surgeries or cardiac catheter interventions, indications for cardiac catheterization leading to collateral vessel closure, peri/post-procedural data and clinical data including the length of ICU and hospital stay were analyzed. To compare the growth of the pulmonary arteries, the Nakata, McGoon and total lower lobe indices, as well as the LPA dimensions normalized for body surface area, were measured angiographically.

Data were analyzed by comparing patients with versus patients without interventional closure of veno-venous collaterals and patients with versus without interventional APC closure after BCPC or/and TCPC. Generative artificial intelligence (GenAI) has not been used in this paper in any way.

### 2.2. Cardiac Catheterization Procedures and Clinical Course

Cardiac catheterizations were performed under general anesthesia with biplane angiography in all procedures. Patients initially received a 100 IU/kg bolus of heparin intravenously. Peri-procedural anticoagulation was monitored by activated clotting time. During cardiac catheterization, veno-venous and aortopulmonary collaterals were identified angiographically by selective contrast injections. For large collaterals, temporary test occlusion was performed to assess changes in systemic venous pressure and oxygen saturation before definitive closure. For smaller vessels, the indication for embolization was based on a subjective angiographic evaluation of contrast flow intensity and washout, complemented by oxygen saturation measurements. Patients who received interventional closure of collateral vessels received antibiotic prophylaxis using a second generation cephalosporin.

Original cardiac anatomy, surgical methods, presence of sildenafil therapy, hospital stay and ICU stay were compared both in patients with and without VVC or APC closure. Previous oxygen levels, invasive hemodynamics and pulmonary artery growth were compared in patients with and without VVC/APC closure (assessed during cardiac catheterization pre BCPC and TCPC). A mean pulmonary artery pressure of ≥ 16 mmHg was defined as elevated, consistent with the original Ten Commandments from Choussat (1977) [19] and the modern criteria that regard high pulmonary pressures as a risk factor for single ventricle circulation failure [20,21,22]. The median follow up was 5.7 (5.4–7.1) years after TCPC.

### 2.3. Statistics

Categorial variables are presented as absolute numbers and percentages. Continuous variables are presented as medians and interquartile ranges or standard deviations, respectively. Statistical analysis was performed using SPSS 29.0.2.0 (SPSS Inc, IBM Company, Chicago, IL, USA). A chi-square test was used for the comparison of categorical data (e.g., patient characteristics in the different groups, VVC closure vs. no VVC closure, APC closure vs. no APC closure). The Mann–Whitney-U test or the univariate Kruskal–Wallis test was used for not normally distributed variables (e.g., comparison of hemodynamic data, PA growth, length of hospital and ICU stay). Significance is defined by values of *p* ≤ 0.05. Given the exploratory nature of the analysis, no correction for multiple comparisons was applied.

### 2.4. Ethics

All data were primarily obtained for medical purposes, with informed consent for the performed cardiac catheterization procedure. The study design fulfills the guidelines of the Declaration of Helsinki regarding ethical principles for medical research involving human subjects. The study was approved by the institutional ethical board (approval number 2017-00564).

## 3. Results

### 3.1. Patient Characteristic and Surgical Procedures

A total of 135 patients were included in the study. Age at first surgical procedure ranged between 0 and 108 days, by a median (Q1–Q3) of 7 days (1.25–49.75). A first surgical procedure after birth was needed in 124 patients (Classic Norwood procedure n = 38; Hybrid-Giessen-approach n = 26; pulmonary artery banding (PAB) n = 26; systemic to pulmonary shunt n = 41). BCPC was performed at a median age of 20.4 weeks (10.25–34.60). TCPC was performed at a median age of 30 months (23.50–46.50). All patients had an extracardiac type of Fontan completion, with a conduit size between 16 to 20 mm, out of which 49 (36.3%) were fenestrated. The complete anatomical characteristics of the patients and surgical/interventional approach are shown in Table 1.

### 3.2. Veno-Venous Collaterals

A total of 40 VVC closures in 34 (25%) patients were performed: 24 (18%) patients after BCPC and 7 (5%) patients after TCPC. Three (2%) patients underwent VVC closure both after BCPC and after TCPC. These three cases had a recanalization of the same VVC that had already been closed before. Six (4%) patients received multiple interventional VVC occlusions. The mean (SD) timing of VVC closure after BCPC was 524 (316) days, with a minimum of 4 days and a maximum of 4475 days. The mean timing of VVC closure after TCPC was 595.5 (1652), with a minimum of 7 days and a maximum of 1547 days. Indications for cardiac catherization after TCPC included invasive reevaluation after diagnosis of VVCs via MRI, residual stenosis of LPA or RPA, persistent need of oxygen supply and reduced systolic function of the single ventricle. Detailed patients’ characteristics were similar between the groups (Table 2). Regarding surgical history, patients with VVC closure had a significantly higher frequency of a comprehensive stage I and II procedure (29.4% vs. 14.8%, *p* = 0.05). They had a higher frequency of a left pulmonary artery patch procedure during TCPC (20.6% vs. 7.9%; *p* = 0.04) and they more often underwent fenestrated Fontan completion during a TCPC procedure (35.7% vs. 27.7%, *p* < 0.001). Patients with embolization of VVCs more often received systemic therapy with Sildenafil (26.5% vs. 14.9%, *p* = 0.13), although this was not statistically relevant.

#### 3.2.1. Description of the VVCs

Most VVCs occurred unilaterally on the left side. The complete description of the origin and draining site of the closed VVCs is shown in Table 3.

#### 3.2.2. Embolization Data

Most VVCs (33/40, 82%) were closed using BALT platinum embolization coils (BALT USA LLC, 29 Parker, Irvine, CA 92618, USA). In 6/40 (15%) VVC closures, Amplatzer Vascular plugs (Abott Amplatzer TM Vascular Plug, AVP) were used [AVP II (8 mm) (n = 3), AVP II (10 mm) (n = 1), AVP I (10 mm) (n = 1), AVP IV (8 mm) (n = 2), AVP I (6 mm) (n = 2)]. In one case (2.5%), a combination of AVP II (8 mm) and BALT coils was used.

#### 3.2.3. Hemodynamics Before BCPC

**Figure 1:**
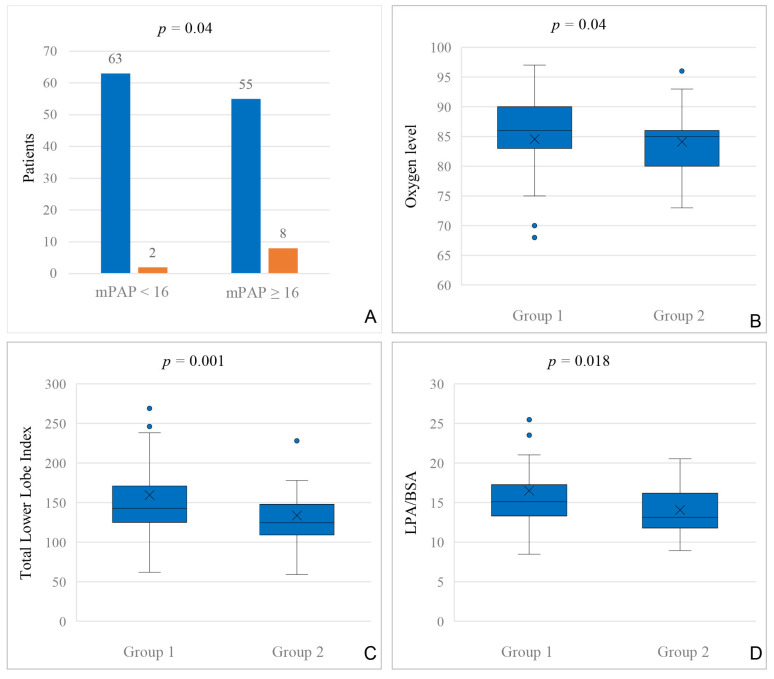
(**A**): Correlation between mean pulmonary artery pressure in mmHg (mPAP) prior to BCPC and the amount of transcatheter VVC closures after total cavopulmonary connection (TCPC); blue = no VVC closure; orange = VVC closure. (**B**): Comparison of oxygen levels (assessed during cardiac catheterization prior to TCPC) in patients without (Group 1) and with (Group 2) VVC closure at any time. (**C**): Total lower lobe index, assessed during cardiac catheterization, prior to TCPC in patients without (Group 1) and with (Group 2) VVC closure. (**D**): LPA/BSA index, assessed during cardiac catheterization, prior to TCPC in patients without (Group 1) and with (Group 2) VVC closure.

Hemodynamic data of patients before BCPC with and without interventional closure of VVCs are shown in Table 4. Mean pulmonary artery pressure (mPAP) >/= 16 mmHg in BCPC patients was associated with a higher incidence of VVC closures after TCPC (*p* = 0.04; Figure 1A).

There was no difference in oxygen levels before BCPC for patients with and without VVC closure.

#### 3.2.4. Hemodynamics Before TCPC

Hemodynamic data of patients before TCPC, with and without interventional closure of VVCs, are shown in Table 4. The mean pulmonary artery pressure (mPAP) >/= 15 mmHg or higher was associated with higher incidence of overall VVC closure (*p* = 0.021). Patients with any type of VVC closure (PV and SV drainage) had a significantly lower oxygen level prior to TCPC [SpO2 level 85% (range 93–73%) vs. 86% (97–76%); *p* = 0.04; Figure 1B].

#### 3.2.5. Oxygen Level Increase

The median (SD) oxygen level increase for patients with VVC closure after BCPC was 4.5% (4.4; *p* = 0.16) and after TCPC it was 5% (0.0; *p* = 0.21). The mean (SD) oxygen level increase after any interventional closure of VVC draining to the PV was 5% (4.72; *p* = 0.27).

#### 3.2.6. Pulmonary Arteries

The size of the pulmonary arteries was assessed by angiography pre-BCPC and pre-TCPC. Patients who received an interventional closure of VVC at any time had a significantly lower Nakata index before BCPC (*p* = 0.03) and a significantly lower total lower lobe index before TCPC (*p* = 0.001; Figure 1C). The LPA/BSA index was significantly lower both before BCPC (*p* = 0.015) and before TCPC (*p* = 0.018; Figure 1D).

All pulmonary artery indices compared in patients with and without VVC closure are shown in Table 5.

#### 3.2.7. Clinical Data

- Hospital stay

Patients with VVC closure at any time had a significantly longer hospital stay, both after BCPC (median 30 days vs. 17 days, *p* = 0.008) and after TCPC (19 days vs. 17 days, *p* = 0.04).

- ICU stay

Patients with VVC closure at any time had a significantly longer need for intensive care after BCPC (ICU stay 6.5 days vs. 5 days; *p* = 0.04) and a trend for longer intensive care after TCPC (ICU stay 4 days vs. 3 days; *p* = 0.13).

### 3.3. Major Aortopulmonary Collaterals

A total of 62 APC closures in 53/135 (39%) patients were performed. A total of 13/135 (9%) had multiple interventional closures of APCs. In totally, 46/135 (34%) underwent APC closure after BCPC and 11/135 (8%) after TCPC. Also, 6/135 (4%) received APC closure both after BCPC and TCPC. The mean (SD) timing of APC closure after BCPC was 542 (320) days, with a minimum of 0 days and maximum of 1663 days. The mean timing of VVC closure after TCPC was 62 (1085), with a minimum of 0 days and a maximum of 3669 days. Indications for cardiac catherization after TCPC included a persistent need for an oxygen supply, persistent high PAP on the ICU, invasive reevaluation of the fenestration and persistent chylous effusion. Patients with single RV more often received embolization of APCs (49.3% vs. 28.1%, *p* = 0.04). Patient characteristics were similar between the groups with and without APC closure (Table 6). Patients with interventional closure of APCs more often underwent a previous Norwood I procedure (37.7% vs. 21.9%; *p* = 0.04) or a comprehensive stage I and II surgery (26,4% vs. 13.4%; *p* = 0.05). They also had an earlier BCPC than patients without APC closure (18.4 weeks vs. 22.4 weeks; *p* = 0.02).

#### 3.3.1. Description of Major Aortopulmonary Collateral Arteries (APCs)

The origin of the APCs was mainly from the internal mammary artery (38/65, 58%), the subclavian artery (19/65, 29%) and the descending aorta (8/65, 12%).

#### 3.3.2. Embolization Data

All embolizations were performed using BALT platinum embolization coils (BALT USA LLC, 29 Parker, Irvine, CA 92618, USA).

#### 3.3.3. Hemodynamics Before BCPC

Hemodynamic data before BCPC of patients, with and without APC closure, are shown in Table 7.

**Figure 2:**
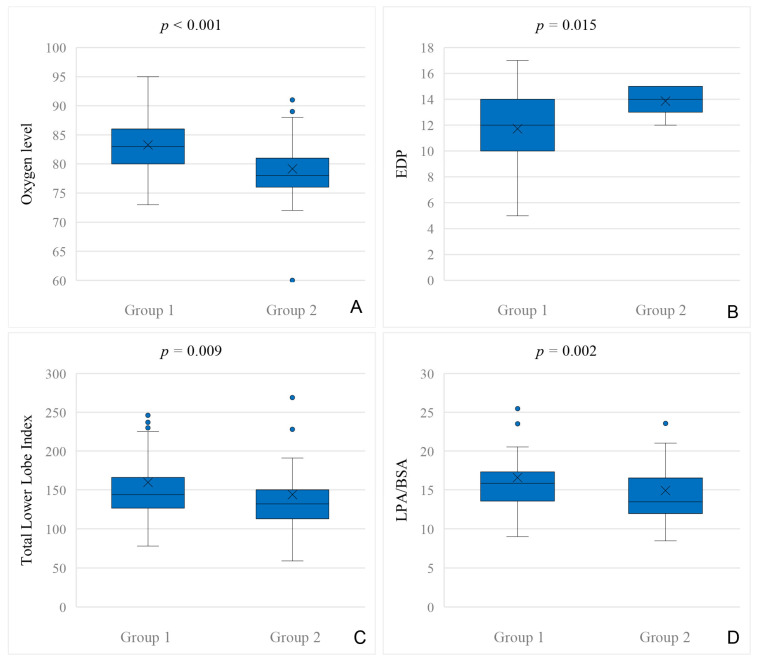
(**A**): Comparison of oxygen levels (assessed during cardiac catheterization, prior to BCPC) in patients without (Group 1) and with (Group 2) APC closure at any time. (**B**): Comparison of the end diastolic pressure (EDP), assessed during cardiac catheterization, pre-TCPC in patients without (Group 1) and with (Group 2) APC closure after TCPC. (**C**): Total lower lobe index, assessed during cardiac catheterization prior to TCPC in patients without (Group 1) and with (Group 2) APC closure. (**D**): LPA/BSA index, assessed during cardiac catheterization prior to TCPC in patients without (Group 1) and with (Group 2) APC closure.

Patients who underwent transcatheter APC closure had significantly lower oxygen levels prior to BCPC (median SpO2 level 78% vs. 83%; *p* < 0.001; Figure 2A).

#### 3.3.4. Hemodynamics Before TCPC

Hemodynamic data before TCPC of patients with and without APC closure are shown in Table 7. Patients who underwent closure of APCs after TCPC (n = 11) had a higher EDP prior to TCPC (*p* = 0.015; Figure 2B).

#### 3.3.5. Pulmonary Arteries

The size of the pulmonary artery vessels was assessed by angiography in the cardiac catheter procedure, pre-BCPC and TCPC. All pulmonary artery indices compared in patients with and without APC closure are shown in Table 8. Patients who underwent transcatheter APC closure had a significantly lower McGoon ratio (*p* < 0.001), Nakata index (*p* = 0.003), total lower lobe index (*p* = 0.009; Figure 2C) and LPA/BSA index (*p* = 0.002; Figure 2D) before TCPC. There was no difference in pulmonary vessel growth indices before BCPC.

#### 3.3.6. Clinical Data

- Hospital stay

Patients with APC closure at any time had a longer hospital stay after TCPC (21 days vs. 15 days; *p* < 0.001) and BCPC (27 days vs. 17 days; *p* = 0.01).

- ICU stay

Patients with an interventional APC closure at any time had a longer need for intensive care after TCPC (5 days vs. 3 days; *p* = 0.005), but not after BCPC (both 5 days; *p* = 0.1). Patients who underwent APC closure after BCPC had a longer ICU stay after TCPC (4.5 days vs. 3 days; *p* = 0.022).

## 4. Discussion

In this single-center retrospective study of 135 patients with single ventricle physiology undergoing staged Fontan palliation, we compared anatomical and clinical outcomes between patients with and without interventional closure of collateral vessels. We found that: (1) veno-venous collaterals (VVCs) and aortopulmonary collaterals (APCs) were frequent findings; (2) their closure was not associated with improved pulmonary artery growth or postoperative outcomes; and (3) fenestration and hemodynamic status influenced the likelihood of VVC closure. These results suggest that collateral closure reflects patient-specific hemodynamic conditions, rather than serving as an isolated therapeutic target.

The wide range in the reported prevalence of collateral vessels (14–31%; 18–85%, respectively) [6,7,8,16,17] likely reflects heterogeneity in definitions, imaging modalities, and timing of evaluation across studies. Some authors include only angiographically relevant vessels, whereas others report all collaterals identified by non-invasive imaging. Differences in patient stage and inclusion of both VVCs and APCs also contribute to the observed variability.

In our cohort, 25% of the patients had transcatheter VVC closure, mostly between BCPC and TCPC. As veno-venous collaterals reduce the effective pulmonary blood flow in single ventricle patients, it is interesting to see their effect on pulmonary artery growth. In our cohort, patients who underwent VVC closure at any time had a lower Nakata index before BCPC compared to patients without VVC closure. Before TCPC, we could not find a difference in Nakata indices in patients with and without VVC closure, but it is of note that in this cohort, stenting of the central PAs between BCPC and TCPC was frequent [23]. In fact, before TCPC, we found a difference in the total lower lobe index (LLI), which bypasses the possible bias generated by a central PA stent. Additionally, there are studies suggesting the use of LLI as a more appropriate measure of pulmonary artery growth, especially after BCPC [24]. As Poterucha et al. discussed, one of the reasons for the formation of veno-venous collaterals can be an anatomical obstruction leading to redirection of the blood flow and formation of VVCs to maintain adequate circulation [6]. In our cohort, we found a significantly lower LPA/BSA index in patients with VVC closure, both before BCPC and TCPC. LPA/BSA is more likely to determine single left-sided pulmonary artery obstructions, as it is calculated—other than the Nakata index—by using only the left pulmonary artery cross sectional area. This indicates a former anatomical, post-surgical, or hemodynamical obstruction on the left side in patients with VVC closure.

In our cohort, the VVCs most frequently occurred unilaterally in the range of the left venous arch. It is important to understand that left-sided VVCs most likely form because of various pre-existing embryologic venous channels, which anatomically occur more often on the left side.

Veno-venous collaterals form in situations with an elevated trans-pulmonary gradient [5] being an expression of a malfunction in the passive pulmonary blood flow after BCPC or TCPC. They thus might form as some sort of natural fenestration [6]. We could not find general hemodynamic differences between patients with and without VVC closure, neither before BCPC nor before TCPC; however, the concomitant use of Sildenafil in a large proportion of patients may have influenced these findings. Our findings are congruent to recently published data from Nguyen Cong et al. [5], who describe similar hemodynamics before TCPC in their cohort of 635 patients with and without veno-venous collaterals.

Sugiyama et al. describe a connection of higher mean pulmonary artery pressure and the diameter of the veno-venous collaterals in patients after TCPC [11]. Interestingly, in our cohort before BCPC, a mPAP of 16 mmHg or higher was found more often in patients who received a VVC closure after TCPC. This might be helpful to predict patients with a higher risk for VVC development already in an early stage and suggests a compromised hemodynamical BCPC status in patients with VVC development [25]. These patients should thus receive a fenestration during TCPC in order to reduce the pulmonary artery pressure and prevent the subsequent formation of VVCs.

Furthermore, patients with interventional closure of VVCs more often had systemic therapy with sildenafil, even if not statistically relevant, and a higher incidence of Fontan fenestration, which might be other indicators for worse hemodynamics. But the observation that patients without fenestration were less likely to undergo VVC closure should be interpreted cautiously. Fenestration was typically performed in patients with less favorable hemodynamics, and this association may therefore reflect underlying physiological differences, rather than a direct effect of VVC closure. It is conceivable that some patients with prior collateral closure experienced hemodynamic worsening, prompting the creation of a surgical fenestration as a controlled decompression pathway. Conversely, fenestration may have served as a standardized and safer substitute for spontaneous venovenous collaterals. These hypotheses remain speculative and warrant further investigation in larger, longitudinal cohorts.

Besides hemodynamic differences in patients with veno-venous collaterals, VVCs are a common cause of cyanosis in single ventricle patients [2,5,10,11]. Ngyuen Cong et al. describe significantly lower oxygen saturation before BCPC in patients that have VVCs closure after BCPC [5], whereas in our study, we could not find different oxygen levels before BCPC for patients with VVC closure after BCPC. In the cardiac catheterization before TCPC, patients with VVC closure had significantly lower oxygen levels. This contrasts with the finding of Nguyen Cong et al., who describe no different oxygen levels pre-TCPC in patients with and without VVCs closure [5].

Although oxygen levels before the closure of VVCs may vary, multiple studies show congruent data on the increase in oxygen levels after the embolization of veno-venous collaterals [5,10,26], which was also the trend for our cohort.

Veno-venous collaterals are known to be associated with longer hospital stays. Ngyuen Cong et al. describe a correlation between VVCs and hospital stay (not ICU stay) [5]. In our study, embolization of veno-venous collaterals was associated with longer overall hospital stay, both after BCPC and TCPC. Besides the length of hospital stay, we also found an association of VVC closure and longer intensive care length of stay, both after BCPC and TCPC. This indicates that those who were embolized are high-risk patients and possibly had borderline hemodynamics, as mean PAP was significantly higher and pulmonary artery growth was significantly lower.

In summary, the decision to close VVCs remains controversial. While closure may improve oxygen saturation and reduce right-to-left shunting, VVCs can act as natural decompression pathways in patients with elevated venous pressures. In such settings, closure may worsen hemodynamics, underscoring the need for careful, individualized assessment. Furthermore, the risk of a recurrence remains high, even after successful closure.

In our cohort, 39% of the patients underwent transcatheter closure of aortopulmonary collaterals. APC closure was more often performed after BCPC (34%) than after TCPC (9%). Beside hypoxia being the main factor for APC growth by stimulating the release of angiogenic factors [15], anatomy or pulmonary artery size have been suggested in previous studies as other aetiologic factors for the development of APCs [16,27]. In our study, APC closure was predominant in single RV patients with a history of a Norwood I procedure or a comprehensive stage I and II. Also, patients in our cohort were younger when they were operated on for BCPC. Schmiel et al. examined associated factors of APC development in 430 single ventricle patients with staged palliation. They also described that APCs were associated with a previous Norwood procedure and a younger age at BCPC [28].

The presence of aortopulmonary collaterals results in a left-to right shunt with a volume load of the pulmonary arteries and the systemic ventricle [2]. Thus, it has been suggested that the presence of APCs might lead to an elevation of PA pressure [29,30]. Nevertheless, there are also studies that show no difference in hemodynamic parameters in association with aortopulmonary collateral formation [31]. We could not find general hemodynamic differences between patients with and without APC closure, neither in cardiac catheterization before BCPC nor before TCPC. The only hemodynamic difference we could find in our data was a higher EDP before TCPC in patients who underwent APC closure after TCPC. Thus, a higher EDP before TCPC might indicate a higher risk for developing APCs after TCPC.

Patients with APC closure had significantly lower oxygen levels prior to BCPC, which is congruent to the findings of Schmiel et al., who describe lower oxygen saturation at Norwood I hospital discharge as an independent risk factor for the development of APCs in HLHS patients [31]. Although, the comparison between this study and our data should be interpreted with caution, both measurements reflect the patient’s condition at a similar stage of single ventricle palliation, provided that no supplemental oxygen was given prior to catheterization.

Although most transcatheter APC closures were performed in patients after BCPC, we could not find a difference in the length of ICU stays after BCPC. Nevertheless, there was an association with longer ICU and hospital stays after TCPC and hospital stays after BCPC. Interestingly, the patients who underwent APC closure after BCPC also had a longer ICU and hospital stay after TCPC. These results might indicate that patients with APCs before TCPC are especially high-risk patients with impaired pulmonary artery perfusion, due to preexistent compromised hemodynamics and reduced pulmonary artery growth. Osawa et al. similarly concluded in their study published in 2023 that there is a correlation between aortopulmonary collaterals before TCPC and prolonged chest tube duration and chylothorax [32]. Also, Grosse-Wortmann et al. describe a correlation between aortopulmonary collaterals and the duration of hospital stay [30]. On the contrary, our results are different to recently published data by Schmiel et al., who describe no difference in ICU stay after TCPC in patients who received interventional closure of APCs before TCPC [28].

The clinical relevance of APCs can be related to their interplay with PA growth, which is a key factor in the outcome of SV patients [23]. Our understanding is that aortopulmonary collaterals form in order to increase pulmonary blood flow [13]. They are known to be associated with insufficient pulmonary vessel growth [28], either because the presence of APCs prevent adequate antegrade perfusion or PA growth was insufficient in the first place and APC formation occurred. The results of our study support this hypothesis, as we found significantly smaller pulmonary arteries in patients who underwent transcatheter APC closure before TCPC compared to the patients who did not. Interestingly, we could not find a difference in pulmonary vessel growth before BCPC. Again, these results are congruent to recently published data: Staehler et al. describe a correlation of aortopulmonary collaterals with a lower pulmonary artery index before TCPC, but this group could not find a significant correlation of these markers at the time before BCPC [14]. Latus et al. have described similar results, as they found a correlation of smaller pulmonary arteries and aortopulmonary collateral flow [33].

Staehler et al. conclude that before BCPC, pulmonary arterial flow might be determined by anatomical features, such as the size of the aortopulmonary shunt or pulmonary stenosis, rather than the pulmonary artery size itself. They conclude from their data that after BCPC, as the passive blood flow via SVC becomes the only pulmonary blood flow, PA size might then become a representative marker for the development of APCs [14]. Our study results support this hypothesis. We also found a correlation of surgical/anatomical features, such as the previously mentioned correlation of a Norwood I or a comprehensive stage I and II procedure with a higher prevalence of APC closure (as explained above). It therefore might be an important factor that PA size is more helpful to predict the development of aortopulmonary collaterals in patients after BCPC, rather than before.

### Limitations

This study is limited by its retrospective and not-randomized, single-center design. The time of development for veno-venous or aortopulmonary collaterals may differ from the time of its transcatheter embolization. Additionally, there is a lack of incorporation and comparison of patients with collateral vessels, where transcatheter closure of collateral vessels was not performed, as the comparison was not based on the occurrence but rather on the interventional embolization of the collateral vessel. The follow-up data after TCPC is relatively short and thus, there is a lack of understanding of the effect of collateral vessel closure in terms of long-time outcome. Longitudinal analysis of hemodynamic trends and their relationship with clinical outcomes would provide valuable additional insights and therefore should be observed in future studies. The comparison of our data with other publications is limited, as indications for interventional closure of VVCs or APCs are individual and vary from center to center.

## 5. Conclusions

From a clinical perspective, our findings emphasize that the indication for interventional closure of collateral vessels in single ventricle patients should be individualized. The presence of veno-venous or aortopulmonary collaterals often reflects the underlying hemodynamic burden, rather than constituting an isolated interventional target. Careful evaluation of ventricular function, conduit size, and systemic venous pressures is essential before deciding on closure. These results underline the importance of comprehensive preoperative assessment and may help refine the selection criteria for collateral embolization in the Fontan pathway.

## Figures and Tables

**Table 1:** Patient characteristics of the complete cohort.

Patients		All Patients	
		n	%
Total		135	100
Sex	Female	56	41.5
	Male	79	58.5
Dominant Ventricle Morphology	Left ventricle	65	48.1
	Right ventricle	70	51.9
Aortic arch	Left	130	96.3
	Right	5	3.7
Diagnosis	Dysbalanced AVSD	10	7.4
	DILV	16	11.9
	DILV, aortic arch anomaly	5	3.7
	DORV	17	12.6
	DORV, aortic arch anomaly	2	1.5
	Ebstein anomaly	1	0.7
	HLHS and HLHS-complex	43	31.9
	PA (VSD or IVS)	18	13.3
	TA	19	14.1
	TA, aortic arch anomaly	1	0.7
	TGA, VSD	3	2.2
**Stage I**		**n**	**%**
Norwood I		38	28.1
	With mBTS	29	21.5
	With AP-Shunt	2	1.4
	With Sano-Shunt	7	5.2
None		11	8.1
Giessen-approach		26	19.2
Central PA-Banding		26	19.2
Shunt	Total	41	30.4
	mBTS	23	17.0
	PDA-Stent	5	3.7
	Aorto-pulmonary-shunt	12	10.4
	Sano shunt	1	5.9
**BCPC**		**median/n**	**range/%**
Age	Weeks	20.43	10.26–34.60
Procedure	BDCPC	102	75.6
	Bilateral BDCPC	9	6.7
	Comprehensive stage I-II	25	18.5
	BDCPC + DKS anastomosis	11	8.1
	Additional PA-Patch	36	26.7
**TCPC**		**median/n**	**range/%**
Age	Months *	30	23.50–46.50
Procedure	ECC	129	95.6
	No TCPC yet/missing data	6	4.4 **
	No fenestration	80	59.3 **
	Fenestration	49	36.3 **
Fenestration	ECC/RA	10	7.4 **
	V.innominata/LA	1	0.7 **
	V.innominata/RA	30	22.2 **
	SVC/RA	8	5.9 **
	Conduit (mm)	18.0	16.0–18.0
Additional PA procedure	LPA patch	15	11.1
	LPA stent (hybrid)	6	4.4
	RPA patch	1	0.7
**Sildenafil**			
	Sildenafil	110 ***	81.5 **
	No Sildenafil	24 ***	17.8 **

Table 1—Characteristics of the complete patients’ cohort, referring to baseline anatomy and procedures at stage I, BCPC and TCPC. Abbreviations—AVSD: atrioventricular septal defect, DILV: double inlet left ventricle, DORV: double outlet right ventricle, HLHS: hypoplastic left heart syndrome, PA: pulmonary atresia, VSD: ventricular septal defect, IVS: intact ventricular septum, TA: tricuspid atresia, TGA: transposition of the great arteries, mBTS: modified Blalock–Taussig shunt, PA: pulmonary arteries, PDA: patent ductus arteriosus, BDCPC: bidirectional cavopulmonary connection, DKS: Damus–Kaye–Stansel, TCPC: total cavopulmonary connection. ECC: extracardiac conduit, RA: right atrium, LA: left atrium, and SVC: superior vena cava. * n = 4 no TCPC yet, n = 2 missing data; ** percentage based on number under line 1 “total”; *** n = 1 missing data.

**Table 2:** Patient characteristics with vs. without VVC closure.

Patients		VVC Closure		No VVC Closure		
		n	% ^†^	n	% ^†^	*p*
Total		34	25.2	101	74.8	
Sex	Female	11	20	43	80	0.21
	Male	23	29.5	55	70.5	
Ventricle	Left ventricle	18	28.5	45	71.5	0.70
	Right ventricle	16	23.2	53	76.8	
Aortic arch	Left	32	94.1	98	97.1	0.44
	Right	2	5.9	3	2.9	
Diagnosis	Dysbalanced AVSD	3	30	7	70	0.71
	DILV	6	37.5	10	62.5	0.22
	DILV, aortic arch anomaly	3	60	2	40	0.06
	DORV	5	29.4	12	70.6	0.66
	DORV, aortic arch anomaly	1	50	1	50	0.41
	Ebstein anomaly	0	0	1	100	0.56
	HLHS and HLHS-complex	8	19	34	81	0.22
	PA (VSD or IVS)	3	17.6	14	82.4	0.37
	TA	4	22.2	14	77.8	0.65
	TA, aortic arch anomaly	1	100	0	0	0.08
	TGA, VSD	0	0	3	100	0.30
**Stage I**		**n**	**%** ^†^	**n**	**%** ^†^	***p***
Norwood I		8	23.5	30	29.7	0.48
	With mBTS	5	14.7	24	23.7	0.26
	With AP-Shunt	1	2.9	1	0.9	0.41
	With Sano-Shunt	2	5.9	5	4.9	0.83
None		2	5.9	9	8.9	0.57
Giessen- approach		10	28.6	16	15.8	0.08
Central PA-Banding		5	14.7	21	20.8	0.43
Shunt						
	mBTS	8	23.5	15	14.8	0.24
	PDA-Stent	1	2.9	4	3.9	0.78
	AP-shunt	4	11.7	8	7.9	0.49
	Sano shunt	0	0	1	0.9	0.56
**BCPC**		**median/n**	**range/%** ^†^	**median/n**	**range/%** ^ † ^	***p***
Age	Weeks	18.92	9.82–43.39	21.00	10.36–31.74	0.39
Procedure	BDCPC	24	70.6	78	77.2	0.43
	Bilateral BDCPC	0	0	9	8.9	0.07
	Comprehensive stage I-II	10	29.4	15	14.8	**0.05**
	BCPC + DKS anastomosis	3	8.8	8	7.9	0.86
	Additional PA-Patch	12	35.3	24	23.7	0.18
**TCPC**		**median/n**	**range/%** ^†^	**median/n**	**range/%** ^†^	***p***
Age	Months	30.00 *	24.40–53.10	30.00 **	22.70–44.00	0.47
Procedure	No fenestration	21	61.7	68	67.3	**<0.001**
	Fenestration	12	35.3	28	27.7	
Fenestration	ECC/RA	4	11.7	6	5.9	0.26
	V.innominata/LA	1	2.9	0	0	0.08
	V.innominata/RA	15	44.1	15	14.8	**0.001**
	SVC/RA	1	2.9	7	6.9	0.39
Conduit	Conduit (mm)	16	16–18	18	16–18	0.13
Additional PA procedure	LPA patch	7	20.6	8	7.9	**0.04**
	LPA stent (Hybrid)	2	5.9	4	3.9	0.63
	RPA patch	0	0	1	0.9	0.56
Sildenafil therapy any time after BCPC						***p***
	Sildenafil	9	26.5	15 ***	14.9	0.13
	No Sildenafil	25	73.5	85 ***	84.1	

Table 2—Comparison of patients with and without VVC closure, referring to baseline anatomy and procedures at stage I, BCPC and TCPC. Abbreviations—AVSD: atrioventricular septal defect, DILV: double inlet left ventricle, DORV: double outlet right ventricle, HLHS: hypoplastic left heart syndrome, PA: pulmonary atresia, VSD: ventricular septal defect, IVS: intact ventricular septum, TA: tricuspid atresia, TGA: transposition of the great arteries, mBTS: modified Blalock–Taussig shunt, PA: pulmonary arteries, PDA: patent ductus arteriosus, BDCPC: bidirectional cavopulmonary connection, DKS: Damus–Kaye–Stansel, TCPC: total cavopulmonary connection. ECC: extracardiac conduit, RA: right atrium, LA: left atrium, SVC: superior vena cava; LPA: left pulmonary artery; and RPA: right pulmonary artery. * n = 1 no TCPC yet; ** n = 3 no TCPC yet, n = 2 missing data; “total”; *** n = 1 missing data; ^†^ percentage based on number under line 1.

**Table 3:** Description of the closed VVCs.

		Closed VVC	
		n	%
Total		40	100
Unilateral	Right sided	4	10
	Left sided	36	90
Bilateral		2	5
Origin	Innominate vein	28	70
	Superior vena cava	2	5
	Azygos/hemiazygos system	4	10
	LSVC	6	15
Connection			
Pulmonary venous system	Total	13	32
	Left pulmonary veins	9	22
	Pulmonary venous atrium	3	7
	Right pulmonary veins	2	5
	Fistula to the right lung	2	5
Systemic venous system	Total	27	68
	Coronary sinus	10	25
	Other systemic veins	17	42

Table 3—Description of the origin and draining of the closed veno-venous collaterals (VVC). LSVC: left superior vena cava and VVC: veno-venous collaterals.

**Table 4:** Hemodynamic data of patients with vs. without VVC closure.

Hemodynamic Parameters	VVC Closure	No VVC Closure	*p*
Pre-BCPC			
Transpulmonary gradient [mmHg]	5 (1.84)	6 (2.13)	0.44
Mean pulmonary artery pressure [mmHg]	16(3.17)	15 (2.78)	0.92
PVRi [Woodunits ×m^2^]	1.6 (0.84)	1.7 (1.62)	0.69
Qp/Qs ratio	0.88 (0.51)	0.99 (0.58)	0.11
EDP [mmHg]	11.5 (3.25)	12 (3.38)	0.92
Pre-TCPC			
Transpulmonary gradient [mmHg]	4 (1.48)	4 (1.19)	0.15
Mean pulmonary artery pressure [mmHg]	14.5 (2.12)	13 (2.13)	0.12
PVRi [Woodunits ×m^2^]	1.4 (0.58)	1.5 (0.51)	0.63
EDP [mmHg]	12.5 (3.06)	12 (2.44)	0.26

Table 4—Hemodynamic data of patients with univentricular congenital heart defects before bidirectional cavopulmonary anastomosis and total cavopulmonary anastomosis. Transcatheter closure of VVC vs. no transcatheter closure of VVC. Data are illustrated as mean (SD). EDP: end diastolic pressure; Qp: pulmonary blood flow; Qs: systemic blood flow; PVRi: index pulmonary vascular resistance; and VVC: veno-venous collaterals.

**Table 5:** Pulmonary vessel growth in patients with vs. without VVC closure.

	VVC	No VVC	p
Before BCPC			
McGoon	1.70 (0.46)	1.80 (0.47)	0.16
Nakata	168 (88.54)	196 (109.38)	**0.03**
Total lower lobe index	137.5 (46.11)	149.0 (52.28)	0.32
LPA/BSA	18.75 (5.37)	21.12 (5.24)	**0.015**
Before TCPC			
McGoon	1.76 (0.33)	1.97 (0.42)	0.09
Nakata	183.5 (77.44)	222.0 (87.00)	0.09
Total lower lobe index	124.5 (33.38)	143.0 (38.23)	**0.001**
LPA/BSA	13.14 (3.00)	15.09 (3.23)	**0.018**

Table 5—Pulmonary vessel growth in patients with univentricular congenital heart defects undergoing Fontan circulation, determined by angiography with pulmonary vascular indices of left and right pulmonary arteries. Data are presented as mean +/− SD.

**Table 6:** Patient characteristics with vs. without APC closure.

Patients		APC Closure		No APC Closure		
		n	% ^†^	n	% ^†^	*p*
Total		53	39.3	82	60.7	
Sex	Female	13	24.5	43	52.4	**0.001**
	Male	40	75.5	39	47.6	
Ventricle	Left ventricle	19	35.8	47	57.3	**0.04**
	Right ventricle	34	64.2	35	42.7	
Aortic arch	Left	52	98.1	78	95.1	0.36
	Right	1	1.9	4	4.9	
Diagnosis	Dysbalanced AVSD	2	3.8	8	9.8	0.19
	DILV	8	15.1	8	9.8	0.05
	DILV, aortic arch anomaly	4	7.5	1	1.2	0.05
	DORV	8	15.1	9	11.0	0.48
	DORV, aortic arch anomaly	1	1.9	1	1.2	0.75
	Ebstein anomaly	0	0	1	1.2	0.42
	HLHS and HLHS-complex	20	37.7	23	28.0	0.23
	PA (VSD or IVS)	4	7.5	15	18.3	0.11
	TA	5	9.4	13	15.9	0.21
	TA, aortic arch anomaly	0	0	1	1.2	0.42
	TGA, VSD	1	1.9	2	2.4	0.83
**Stage I**		**n**	**%** ^†^	**n**	**%** ^†^	***p***
Norwood I		20	37.7	18	21.9	**0.04**
	With mBTS	14	26.4	15	18.3	0.26
	With AP-Shunt	1	1.9	1	1.2	0.75
	With Sano-Shunt	5	9.6	2	2.4	0.07
None		2	3.8	9	11.0	0.13
Giessen-approach		14	26.9	12	14.6	0.09
Central PA-Banding		10	18.9	16	19.5	0.92
Shunt						
	mBTS	7	13.2	16	19.5	0.34
	PDA-Stent	1	1.9	4	4.9	0.36
	Aorto-pulmonary-shunt	3	5.7	9	11.0	0.28
	Sano shunt	0	0	1	1.2	0.42
**BCPC**		**median/n**	**range/%** ^†^	**median/n**	**range/%** ^†^	***p***
Age	Weeks	18.43	10.10–38.97	22.43	11.02–33.85	**0.02**
Procedure	BDCPC	37	69.8	65	79.2	0.21
	Bilateral BDCPC	2	3.8	7	8.5	0.27
	Comprehensive stage I-II	14	26.4	11	13.4	**0.05**
	BDCPC + DKS anastomosis	3	5.7	8	9.8	0.39
	Additional PA-Patch	16	30.2	20	24.4	0.45
TCPC		**median/n**	**range/%** ^†^	**median/n**	**range/%** ^†^	***p***
Age	months	30 *	20.00–46.70	31 **	24.00–47.20	0.09
Procedure	No fenestration	31	58.5	49	59.8	0.64
	Fenestration	21	39.6	28	34.1	
Fenestration	ECC-RA	5	9.4	5	6.1	0.47
	V.innominata/LA	0	0	1	1.2	0.42
	V.innominata/RA	15	28.3	15	18.3	0.17
	SVC/RA	1	1.9	7	8.5	0.11
Conduit	Conduit (mm)	18	16–18	18	16–18	0.41
Additional PA procedure	LPA patch	7	13.2	8	9.75	0.53
	LPA stent (Hybrid)	4	7.4	2	2.44	0.16
	RPA patch	0	0	1	1.22	0.42
Sildenafil therapy						***p***
	Sildenafil	11	20.37	13	15.85	0.43
	No Sildenafil	41	75.93	69	84.15	

Table 6—Comparison of patients with and without APC closure, referring to baseline anatomy and procedures at stage I, BCPC and TCPC. Abbreviations—AVSD: atrioventricular septal defect, DILV: double inlet left ventricle, DORV: double outlet right ventricle, HLHS: hypoplastic left heart syndrome, PA: pulmonary atresia, VSD: ventricular septal defect, IVS: intact ventricular septum, TA: tricuspid atresia, TGA: transposition of the great arteries, mBTS: modified Blalock–Taussig shunt, PA: pulmonary arteries, PDA: patent ductus arteriosus, BDCPC: bidirectional cavopulmonary connection, DKS: Damus–Kaye–Stansel, TCPC: total cavopulmonary connection. ECC: extracardiac conduit, RA: right atrium, LA: left atrium, SVC: superior vena cava; LPA: left pulmonary artery; and RPA: right pulmonary artery. * n = 1 no TCPC yet; ** n = 3 no TCPC yet, n = 2 missing data; ^†^ percentage based on number under line 1 “total”.

**Table 7:** Hemodynamic data of patients with vs. without APC closure.

Hemodynamic Parameters	APC Closure	No APC Closure	*p*
Pre-BCPC			
Transpulmonary gradient [mmHg]	6.00 (1.86)	5.00 (2.18)	0.16
Mean pulmonary artery pressure [mmHg]	16.00 (2.83)	15.00 (2.89)	0.29
PVRi [Woodunits ×m^2^]	1.70 (0.81)	1.68 (1.79)	0.44
Qp/Qs ratio	0.90 (0.73)	0.99 (0.45)	0.43
EDP [mmHg]	11.00 (3.56)	12.00 (3.21)	0.78
Pre-TCPC			
Transpulmonary gradient [mmHg]	4.00 (1.25)	4.00 (1.21)	0.55
Mean pulmonary artery pressure [mmHg]	13.00 (2.22)	14.00 (2.25)	0.89
PVRi [Woodunits ×m^2^]	1.48 (0.52)	1.50 (0.54)	0.46
EDP [mmHg]	12.00 (2.85)	12.00 (2.58)	0.93

Table 7—Hemodynamic data of patients with univentricular congenital heart defects before BCPC and before TCPC. Transcatheter closure of APCs vs. no transcatheter closure of APCs. Data are presented as the mean (SD). EDP: end diastolic pressure; Qp: pulmonary blood flow; Qs: systemic blood flow; PVRi: index pulmonary vascular resistance; and APC: major aortopulmonary collaterals.

**Table 8:** Pulmonary vessel growth in patients with vs. without APC closure.

	APCs	No APCs	p
Before BCPC			
McGoon	1.70 (0.48)	1.83 (0.47)	0.17
Nakata	170.00 (110.57)	196.50 (103.51)	0.08
Total lower lobe index	144.00 (48.19)	150.50 (52.66)	0.62
LPA/BSA	19.70 (6.18)	21.15 (4.80)	0.14
Before TCPC			
McGoon	1.69 (0.36)	2.00 (0.39)	**<0.001**
Nakata	194.00 (73.64)	228.00 (88.48)	**0.003**
Total lower lobe index	132.00 (39.38)	144.00 (36.27)	**0.009**
LPA/BSA	13.46 (3.25)	15.84 (3.05)	**0.002**

Table 8—Pulmonary vessel growth in patients with univentricular congenital heart defects undergoing Fontan circulation, determined by angiography with pulmonary vascular indices of the left and right pulmonary arteries. Data are illustrated as mean +/− SD.

## Data Availability

The authors confirm that the data supporting the findings of this study are available within the article.

## Abbreviations

The following abbreviations are used in this manuscript:

SV	Single ventricle
VVCs	Veno-venous collaterals
APCs	Major aortopulmonary collaterals
PA	Pulmonary artery
LPA	Left pulmonary artery
(m)PAP	(Mean) Pulmonary artery pressure
BCPC	Bidirectional cavopulmonary connection
TCPC	Total cavopulmonary connection
ICU	Intensive care unit
EDP	End diastolic pressure
PV	Pulmonary veins
SV	Systemic veins
IVC	Inferior vena cava
GenAI	Generative artificial intelligence
PAB	Pulmonary artery banding
SD	Standard deviation
BSA	Body surface area
RV	Right ventricle
(T)LLI	(Total) lower lobe index
